# Gradient Boosting Decision Tree-Based Method for Predicting Interactions Between Target Genes and Drugs

**DOI:** 10.3389/fgene.2019.00459

**Published:** 2019-05-31

**Authors:** Ping Xuan, Chang Sun, Tiangang Zhang, Yilin Ye, Tonghui Shen, Yihua Dong

**Affiliations:** ^1^School of Computer Science and Technology, Heilongjiang University, Harbin, China; ^2^School of Mathematical Science, Heilongjiang University, Harbin, China

**Keywords:** drug–target interaction prediction, class imbalance, ensemble learning, path category-based features, gradient boosting decision tree

## Abstract

Determining the target genes that interact with drugs—drug–target interactions—plays an important role in drug discovery. Identification of drug–target interactions through biological experiments is time consuming, laborious, and costly. Therefore, using computational approaches to predict candidate targets is a good way to reduce the cost of wet-lab experiments. However, the known interactions (positive samples) and the unknown interactions (negative samples) display a serious class imbalance, which has an adverse effect on the accuracy of the prediction results. To mitigate the impact of class imbalance and completely exploit the negative samples, we proposed a new method, named DTIGBDT, based on gradient boosting decision trees, for predicting candidate drug–target interactions. We constructed a drug–target heterogeneous network that contains the drug similarities based on the chemical structures of drugs, the target similarities based on target sequences, and the known drug–target interactions. The topological information of the network was captured by random walks to update the similarities between drugs or targets. The paths between drugs and targets could be divided into multiple categories, and the features of each category of paths were extracted. We constructed a prediction model based on gradient boosting decision trees. The model establishes multiple decision trees with the extracted features and obtains the interaction scores between drugs and targets. DTIGBDT is a method of ensemble learning, and it effectively reduces the impact of class imbalance. The experimental results indicate that DTIGBDT outperforms several state-of-the-art methods for drug–target interaction prediction. In addition, case studies on *Quetiapine, Clozapine, Olanzapine, Aripiprazole*, and *Ziprasidone* demonstrate the ability of DTIGBDT to discover potential drug–target interactions.

## Introduction

Computational prediction of drug–target interactions (DTIs) plays a key role in drug discovery and repositioning (Chen et al., 2015; Yu et al., 2015, 2017b). Drugs exert their functions by interacting with various targets, of which genes are one important group. Through binding, drugs can either enhance or inhibit the expressions of genes and thereby affect disease processes (Overington et al., 2006; Yu et al., 2016; Santos et al., 2017). However, in most cases, drugs may cause multiple side-effects because they can interact with several unintended targets. The identification of targets that interact with drugs by biological and chemical experiments is very laborious and expensive (Langley et al., 2017). Therefore, many studies have attempted to predict DTIs by using computational methods, to reduce the workload and costs in providing candidate DTIs for biologists to verify (Ding et al., 2017a,b, 2019; Shen et al., 2017).

Several prediction methods concentrate primarily on incorporating information from drug–target homogeneous networks (Mei et al., 2012; Xu et al., 2014a,b, 2016; Li et al., 2015; Hao et al., 2017; Yu et al., 2017a). For example, Bleakley and Yamanishi constructed a support vector machine (SVM) framework named BLM, which is based on a bipartite local model, to predict DTIs (Bleakley and Yamanishi, 2009). However, because this method is trained with a large-scale bipartite graph model, high computational power is needed. Mei et al. analyzed DTI features from neighbors and predicted novel interactions (Mei et al., 2012); it is difficult to obtain enough neighbor information for this method. Ezzat et al. and Luo et al. incorporated topological information by applying a random walk on the homogeneous network and used graph regularized matrix factorization to calculate the propensities of DTIs (Ezzat et al., 2017; Luo et al., 2017). However, the accuracy of the results may be influenced when the features are projected into low-dimensional space, because some valuable information may be lost. Hao et al. proposed a method based on non-linear integral of similarity measurements (Hao et al., 2017). Although this method showed good performance, its accuracy depended heavily on the similarity measurements. DTI prediction has been treated as a binary classification problem in Lee's methods (Lee and Nam, 2018). The features of drugs and targets that were used for training a *k*-nearest-neighbors model were weighted by random walks. However, the known and unknown DTIs have a serious class imbalance, which has an adverse impact on prediction accuracy. In DDR, which was applied by Olayan et al., path category-based feature vectors were constructed to incorporate the topological information of the network, and a random forest was used for DTI prediction (Olayan et al., 2017). Random forest does not perform as well as in classification when it solves the regression problem, because it cannot yield a continuous output.

In this work, in order to further improve the accuracy of DTI prediction and mitigate the impact of class imbalance, we propose a novel computational method named DTIGBDT. We construct a drug–target heterogeneous network to extract features. A gradient boosting decision tree (GBDT)-based prediction model is used for calculating the propensities of interactions. We compare our approach with other prediction methods using various performance measurements: the results show that DTIGBDT outperforms the other methods.

## Materials and Methods

Our goal is to predict novel (that is, unknown) interactions between drugs and targets. In order to integrate the information of various connections and the node attributes, we construct a drug–target heterogeneous network. We then design a novel prediction model based on GBDT for the network, to obtain the interaction scores of drug–target pairs. The higher the score, the more likely they are to interact (Zou et al., 2015; Zeng et al., 2017a).

### Dataset for DTI Prediction

We obtained the drug–target interaction data from a published work (Luo et al., 2017). In this dataset, there are 1923 known DTIs, involving 708 drugs from DrugBank 5.0 (Wishart et al., 2017) and 1,412 targets from HPRD 9.0 (Keshava Prasad et al., 2008). For each pair of drugs and each pair of targets, we also extracted the similarities between them from these two databases. The similarity between two drugs was calculated by using the Tanimoto coefficient (Francesco et al., 2010), based on their chemical structures. The similarity between two targets is measured by the Smith-Waterman score (Wenhui et al., 2014), based on their primary sequences.

### Heterogeneous Network-Based Feature Extraction

#### Construction of Drug–Target Heterogeneous Network

**Figure 1:**
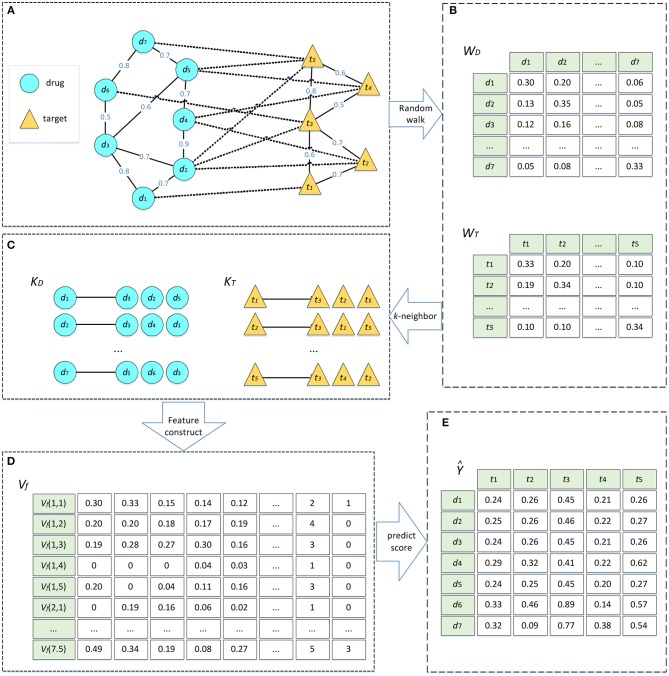
Algorithm flow of DTIGBDT. **(A)** Construct the heterogeneous network. **(B)** Random walk on drug network and target network, respectively. **(C)** Select most similar *k* neighbors. **(D)** Get feature vectors for each drug–target pair. **(E)** Train the DTIGBDT with the feature vectors.

We defined a set of DTIs, which consists of a set of drugs *D* and a set of targets *T*, where *D* = {*d*_1_*, d*_2_,…, *d*_*m*_} includes *m* drug nodes, and *T* = {*t*_1_, *t*_2_,…, *t*_*n*_} contains *n* target nodes. The drug–target network can be considered as a heterogeneous network, which is constructed by a drug network and a target network. In these two networks, we added an edge to connect two drug nodes or two target nodes when the similarity between them were >0. Furthermore, the edge was weighted by the similarity between the two nodes. The edge between a drug and a target represented a known DTI and was weighted by 1. This heterogeneous network can be represented as in Figure 1A.

The interactions between *D* and *T* could also be represented as a matrix *Y* where *Y*_*ij*_ is 1 if drug *d*_*i*_ and target *t*_*j*_ are observed to interact and 0 otherwise. The set of similarities between drugs was represented by *S*_*D*_ϵ*R*^m^*^m^ and the set of similarities between targets was represented by *S*_*T*_ϵ*R*^n^*^n^. The element values in *S*_*D*_ or *S*_*T*_ are in the range of [0, 1] which represents how similar drugs or targets are to each other.

#### Similarity Calculation Based on Network and Selection of k Neighbors

Random walk with restart, a network diffusion algorithm, has been widely used to analyze complex biological network data (Köhler et al., 2008; Tong et al., 2008; Berger et al., 2010; Li and Patra, 2010; Xu et al., 2016; Cheng et al., 2018b; Gao et al., 2018). Random walk can consider the topological information of the network to fully analyze the potential associations between nodes. We conduct random walks on the drug and target networks separately, to extract the topological information of the networks. Based on these similarities, we select the *k* most similar neighbors for each node.

We take the drug network as an example to illustrate the random walk procedure. We defined a matrix *N*_*D*_, in which each element *N*_*D*_ (*i, j*) describes the probability of a transition from *d*_*i*_ to *d*_*j*_.

(1) $$\begin{array}{l} N_{D}\left(i,j\right)=\frac{S_{D}\left(i,j\right)}{\sum_{j^{\prime }}S_{D}\left(i,j^{\prime }\right)} \end{array}$$

where *S*_*D*_(*i, j*) represents the similarity between two drugs, *d*_*i*_ and *d*_*j*_. Next, we defined a matrix $W_{D}^{t}$ϵ*R*^m^*^m^ where $W_{D}^{t}\left(i,j\right)$ is the probability that the walker reaches *d*_*j*_ from *d*_*i*_ after *t* iterations in the random walk process. The matrix *W*_*D*_*t* can be calculated as Equation (2).

(2) $$\begin{array}{l} W_{D}^{t+1}=\left(1-a\right)N_{D}W_{D}^{t}+aW_{D}^{0} \end{array}$$

where parameter *a* is the restart probability. The matrix $W_{D}^{0}$ can be initialized by Equation (3).

(3) $$\begin{array}{l} W_{D}^{0}\left(i,j\right)=\{\begin{array}{c} 1,\;i=j\\ 0,\;i\neq j \end{array} \end{array}$$

The convergence condition of the random walk procedure is $∥W_{D}^{t}-W_{D}^{t-1}{∥}_{1}<10^{-6}$. After the condition is satisfied, the converged probability $W_{D}^{t}\left(i,j\right)$ can be regarded as a similarity score between two drugs. This score incorporates the topological information in the drug network and is used to update the weight of the edge between *d*_*i*_and *d*_*j*_. Next, we selected the *k* most similar neighbors of *d*_*i*_ based on the similarities. We obtained the matrix *K*_*D*_ϵ*R*^m^*^k^ where the *i*th row stores the *k* most similar neighbors of *d*_*i*_. Similarly, we conducted random walk on the target network to obtain the similarity matrix $W_{T}^{t}\left(i,j\right)$ϵ*R*^n^*^n^ and the matrix of the *k* most similar neighbors, *K*_*T*_ϵ*R*^n^*^k^ (Figures 1B,C).

#### Path Category-Based Features

Based on the assumption that similar drugs can usually interact with the same target and vice versa, we extracted an 18-dimensional feature vector based on the path category for each drug–target pair. In this study, we worked with the path categories whose lengths are 2 and 3 (but not longer than that, because of the computational cost). If we limit paths to start at the drug nodes and end at the target nodes, there are only two path categories with length 2. These two categories can be denoted as *C*_1_: (D–D–T) and *C*_2_: (D–T–T), where *D* represents a drug node and *T* represents a target node. The four categories with paths of length 3 are *C*_3_ :(D–T–T–T), *C*_4_ :(D–D–T–T), *C*_5_ :(D–D–D–T), and *C*_6_ :(D–T–D–T). We considered these six categories of paths to predict whether the drug can interact with the target. In this process, we started from a given drug *d*_*i*_ to reach a given target *t*_*j*_ through a specific path category *C*_*h*_, where *h* is selected from {1, 2, 3, …, 6}. We only considered paths that pass through the *k* nearest neighbors of *d*_*i*_ or *t*_*j*_. We denoted the set of such paths as *R*_*ijh*_. Next, for the *q*th path *p*_*q*_ between *d*_*i*_ and *t*_*j*_, we calculated a weight *s* by multiplying all weights on the edges of path *p*_*q*_ as Equation (4).

(4) $$\begin{array}{l} s\left(i,j,h,q\right)=\underset{\forall e_{x}\in p_{q}}{\prod }w_{x} \end{array}$$

where *e*_*x*_is the *x*th edge of *p*_*q*_, and *w*_*x*_ is the weight of the edge. We defined three matrices *V*_1_ϵ*R*^i^*^j^*^h^, *V*_2_ϵ*R*^i^*^j^*^h^, and *V*_3_ϵ*R*^i^*^j^*^h^, to store the features between *d*_*i*_ and *t*_*j*_ under each path category *C*_*h*_. *V*_1_(*i, j, h*) is the sum of the *s*-values in set *R*_*ijh*_. *V*_2_(*i, j, h*) is the maximum *s*-value in set *R*_*ijh*_, and *V*_3_(*i, j, h*) is the number of paths in the set.

(5) $$\begin{array}{l} V_{1}\left(i,j,h\right)=\underset{\forall p_{q}\in R_{ijh}}{\sum }s\left(i,j,h,q\right) \end{array}$$

(6) $$\begin{array}{l} V_{2}\left(i,j,h\right)=\underset{\forall p_{q}\in R_{ijh}}{\max}\left(s\left(i,j,h,q\right)\right) \end{array}$$

(7) $$\begin{array}{l} V_{3}\left(i,j,h\right)=num_{\forall p_{q}\in R_{ijh}}\left(p\right) \end{array}$$

We combined the three matrices into a new matrix *V*_*f*_ϵ*R*^i^*^j^*^(3^*^h)^, where the row *V*_*f*_ *(i, j)* represents the feature vector of *d*_*i*_ and *t*_*j*_ (Figure 1D).

**Figure 2:**
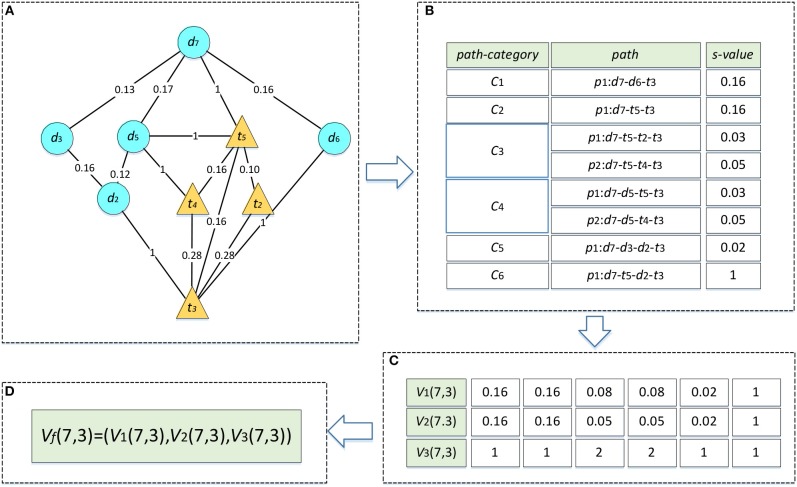
Feature vector calculation of *d*_7_-*t*_3_. The edges between drug nodes or target nodes are weighted by the similarities between two nodes. The edges between drugs and target nodes represent the known DTIs and are weighted by 1. **(A)** Paths between d_7_ and t_3_. **(B)** The *s*-values of all the paths. **(C)** Three types of path feature vectors. **(D)** Connection of three feature vectors.

We take the drug–target pair (*d*_7_, *t*_3_) in Figure 1A as an example to describe the process of heterogeneous network-based feature extraction. The paths from *d*_7_ to *t*_3_ are shown in Figure 2A, and the values of *s* for each path are listed in Figure 2B. There are two paths in the set *R*_733_, *p*_1_: *d*_7_-*t*_5_-*t*_2_-*t*_3_ and *p*_2_: *d*_7_-*t*_5_-*t*_4_-*t*_3_, and the values of *s* for these paths are 0.03 and 0.05, respectively. *V*_1_(7,3,3) is set as the sum of these *s-*values, 0.08. *V*_2_(7,3,3) is set as the maximum of them, 0.05. *V*_3_(7,3,3) is set as the number of the paths, 2.

In terms of the fifth type of path categories *C*_5_, there is only one path *p*_1_: *d*_7_-*d*_3_-*d*_2_-*t*_3_ in the set *R*_735_, and the *s* of *p*_1_ is 0.02. Therefore, *V*_1_(7,3,5) and *V*_2_(7,3,5) are both set as 0.02 and *V*_3_(7,3,5) is set as 1. Similarly, we can compute the features for the other path categories. As a result, the rows which represent the feature vectors of (*d*_7_, *t*_2_) in matrix *V*_1_, *V*_2_, *V*_3_ are set as (0.16, 0.16, 0.08, 0.08, 0.02, 1), (0.16, 0.16, 0.05, 0.05, 0.02, 1), and (1, 1, 2, 2, 1, 1), respectively (Figure 2C). Finally, these three vectors are combined into a single vector of *V*_*f*_, namely *V*_*f*_ (7,3) (Figure 2D).

### DTI Prediction Model Based on GBDT

In our dataset, there are only 1,923 known drug–target interactions, while more than 300,000 interactions are unknown, which causes a serious class imbalance. Aiming to reduce the impact of class imbalance and make full use of the negative samples in the dataset, we constructed an ensemble learning model based on GBDT (Ye et al., 2009), and refer to it as DTIGBDT.

The feature of a drug–target pair (*d*_*i*_, *t*_*j*_) is denoted by a vector *V*_*f*_*(i, j)*. Let *X*_*i,j*_ = {*x*_1_,*x*_2_…,*x*_*z*_} represent *z* subsets of *V*_*f*_(*i, j*), *x*_*k*_ was obtained by randomly sampling some of the features from *V*_*f*_*(i, j)*. For each element in *X*_*i,j*_, we built a decision tree model that is used for predicting the potential DTIs. In this way, we obtained a set *T*_*i,j*_ = {*T*_1_, *T*_2_…, *T*_*z*_} that denotes *z* decision trees. Finally, we obtained the interaction score of the pair by summing the score of all decision trees. This can be calculated as Equation (8).

(8) $$\begin{array}{l} score\left(i,j\right)=\frac{1}{z}\overset{z}{\underset{k=1}{\sum }}\lambda_{k}T_{k}\left(x_{k}\right) \end{array}$$

where *T*_*k*_(*x*_*k*_) represents the score of the decision tree *T*_*k*_. λ_*k*_ is used to adjust the contribution of *T*_*k*_. The greater the value of *score*(*i, j*), the more likely *d*_*i*_ is to interact with *t*_*j*_. We thereby obtained a matrix Ŷϵ*R*^m^*^n^ where Ŷ_*ij*_ = *score* (*i, j*) (Figure 1E). We used the negative log-likelihood to calculate the loss of DTIGBDT.

(9) $$\begin{array}{l} loss=\underset{i,j}{\sum }\log\left(1+\text{exp}\left(-2Y_{ij}{\hat{Y}}_{ij}\right)\right) \end{array}$$

where *Y*_*i,j*_ is the actual interaction between *d*_*i*_ and *t*_*j*_. We defined the objective function as Equation (10).

(10) $$\min\begin{array}{c} L\left(\hat{Y}\right)=loss+\text{λ}\left\|\hat{Y}\right\| \end{array}$$

**Figure 3:**
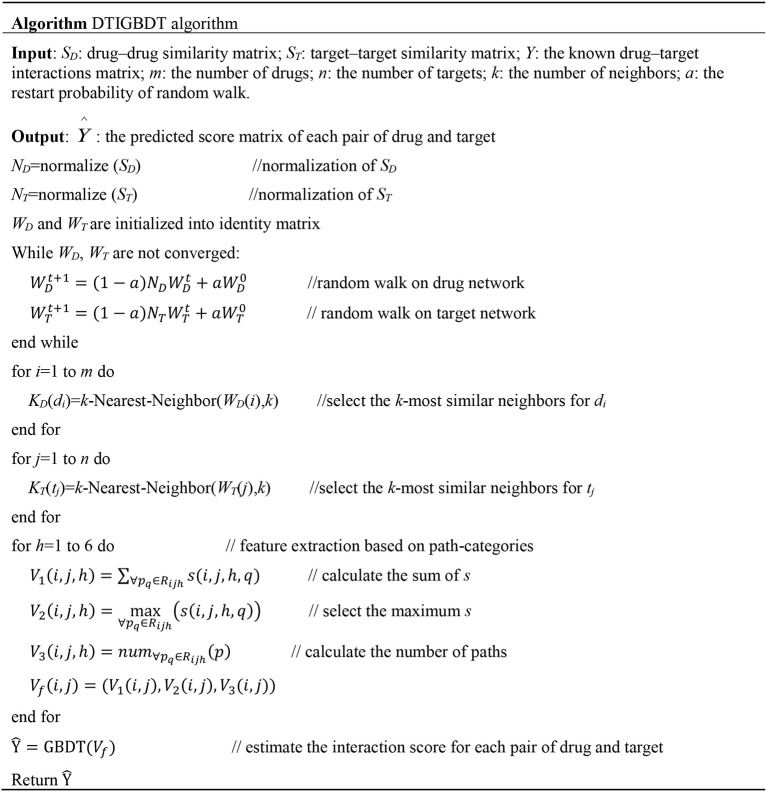
Algorithm for predicting the potential drug–target interactions.

The first term is the loss of DTIGBDT. The second term is the regular term to prevent overfitting, and λ is the regularization parameter for adjusting this term's contribution. The converged Ŷ is the interaction score matrix, which can be calculated by Figure 3.

## Experimental Evaluation and Discussion

### Performance Evaluation Metrics

To evaluate our method and the state-of-the-art methods for DTI prediction, we performed five-fold cross validation (Cheng et al., 2015; Chen et al., 2017; Lin et al., 2017; Wei et al., 2017a, 2018; Zeng et al., 2017b; Bu et al., 2018; Su et al., 2018; Xu et al., 2018b,c). All known DTIs were randomly divided into five subsets with equal size, and the same operation was applied to the unknown interactions (Liu et al., 2017; Zhang et al., 2017; Zeng et al., 2018). In each cross-validation trial, a subset of known DTIs and another subset of unknown DTIs were selected in turn as the test set, while the remaining DTIs were used for training a prediction model. The known and unknown interactions were regarded as the positive and negative samples, respectively. After the prediction is performed, each sample was given a predicted score which represents the propensity of the drug to interact with the target. The positive and negative samples were ranked by their score. The higher the positive samples were ranked, the better was the prediction performance.

For a given threshold δ, if the score of a positive sample was >δ, it was considered as a true positive sample (TP), and if the score was <δ, it would be considered as a false negative sample (FN). If the score of a negative sample was lower than δ, it would be regarded as a true negative sample (TN). If the score was <δ, it would be regarded as a false positive sample (FP). We obtained a receiver operating characteristic (ROC) curve (Streiner and Cairney, 2007) by calculating the true positive rates (*TPRs*) and false positive rates (*FPRs*) for various values of δ.

(11) $$\begin{array}{l} TPR=\frac{TP}{TP+FN}FPR=\frac{FP}{TN+FP} \end{array}$$

The areas under the ROC curves (AUCs) were used to evaluate the performance of each method (Lobo et al., 2008; Cheng et al., 2014, 2018a; Dao et al., 2018; Feng et al., 2018; Nie et al., 2018; Tang et al., 2018; Xu et al., 2018a; Yang et al., 2018). It is generally believed that the closer the value of AUC is to 1, the better the performance is. However, in the case of imbalanced data, AUPR (the area under the precision–recall curve) can provide a more valuable metric (van Laarhoven et al., 2011; Saito and Rehmsmeier, 2015; Patel et al., 2017; Sahiner et al., 2017; Wei et al., 2017b; Jiang et al., 2018a,b). Therefore, we also used AUPR as another measurement to evaluate the performance of each method. The precision–recall curve was constructed by precision rates and recall rates, which are defined as Equation (12).

(12) $$\begin{array}{l} Precision=\frac{TP}{TP+FP}\;Recall=\frac{TP}{TP+FN} \end{array}$$

In addition, biologists usually select the top section of the prediction result for a wet-lab experiment to further validate. As a result, the accuracy of the top *k* candidates is more important for discovering novel DTIs. We demonstrate the recall rates within the top *k* (*k* = 50, 100, 150, 200, 250, 300) candidates to reveal how many of these positive samples are identified successfully.

### Comparison With Other Methods

We compared DTIGBDT with four state-of-the-art methods for DTI prediction, including GRMF (Ezzat et al., 2017), DTINet (Luo et al., 2017), Lee's method (Lee and Nam, 2018), and DDR (Olayan et al., 2017). We describe these methods in more detail below.

**GRMF:** This method proposed a matrix factorization-based model to predict novel DTIs. The drug–target interaction matrix **Y** were decomposed into two low-rank latent feature matrices **A** (for drugs) and **B** (for targets) by using the SVD algorithm. Alternating least squares was used to iteratively update A and B. The optimization problem can be described as:

(13) $$\begin{array}{l} \;\underset{A,B}{\min}{\left\|Y-AB^{T}\right\|}_{F}^{2}\\ +\lambda_{l}\left({\left\|A\right\|}_{F}^{2}+{\left\|B\right\|}_{F}^{2}\right)\\ \;+\lambda_{d}Tr\left(A^{T}{\tilde{L}}_{d}A\right)\\ \;+\lambda_{t}Tr\left(B^{T}{\tilde{L}}_{t}B\right) \end{array}$$

where ${\tilde{L}}_{d}$ and ${\tilde{L}}_{t}$ are the normalized graph Laplacians that were computed based on the similarities between drugs or targets. λ_*l*_, λ_*d*_, and λ_*t*_ are parameters that adjust the contribution of the terms. The interaction score Ŷ_*i,j*_ of drug *d*_*i*_ and target *t*_*j*_ can be calculated as:

(14) $$\begin{array}{l} \;{\hat{Y}}_{i,j}=a_{i}b_{j}^{T} \end{array}$$

where *a*_*i*_ is the *i*th row of A and *b*_*j*_ is the *j*th row of B.

**DTINet:** Heterogeneous data sources provide diverse information for DTI prediction, so Luo et al. integrated four types of drug similarities and three types of target similarities. The random walk with restart algorithm was applied to extract the topological information of the drug network and the target network, and the result of the algorithm was a matrix *S*_*D*_. The low-rank model $S_{D}\approx XW^{T}$ used *X* to represent the corresponding low-dimensional feature vector of each drug. Similarly, the low-dimensional feature vectors of targets could be calculated and were represented by a matrix *Y*. Let *P* denote the interactions between drugs and targets; matrix *Z* can then be calculated by Equation (15).

(15) $$\begin{array}{l} XZY^{T}\approx P \end{array}$$

The interaction score between drug *d*_*i*_ and target *t*_*j*_ was defined as follows:

(16) $$\begin{array}{l} \text{score}\left(i,j\right)=x_{i}Zy_{j}^{T} \end{array}$$

where *x*_*i*_ is the *i*th row of *X* and is the feature vector of *d*_*i*_, and *y*_*j*_ is the *j*th row of *Y* and is the feature vector of *t*_*j*_.

**Lee's method:** In this method, each drug was represented by a bit vector, in which each bit suggests whether a specific sub molecular structure is contained by the drug. In addition, Lee et al. constructed a model based on random walk with restart to extract the topological information of the drug–drug interaction network. The rows of the matrix *F*^*d*^ were used to store the bit vectors of each drug and a matrix *N*^*d*^ was defined to denote the result of the random walk. The final representation of drug *d*_*i*_, denoted by $\nu_{i}^{d}$, was calculated by Equation (17):

(17) $$\begin{array}{l} \nu_{i}^{d}=N_{i}^{d}*F_{i}^{d} \end{array}$$

where $N_{i}^{d}$ and $F_{i}^{d}$ are the *i*th row of *N*^*d*^ and *F*^*d*^, respectively. Similarly, Lee et al. can calculate a vector $\nu_{j}^{t}$ to represent the target *t*_*j*_. The feature vector of the drug–target pair (*d*_*i*_, *t*_*j*_) can be obtained by connecting $\nu_{i}^{d}$ and $\nu_{j}^{t}$. On the basis of the Euclidean distance between each pair of drug and target, a *k*-nearest-neighbor model was trained to infer whether a target interacted with the drug.

**DDR**: DDR constructed a drug-target heterogeneous graph that contains the known DTIs with multiple drug similarities and target similarities. A non-linear similarity fusion method was performed to obtain the optimized drug similarities and the target similarities. For each drug–target pair, DDR constructed a path-category-based feature, which integrates the sum of the paths' weight and the maximum weight of the paths. A random forest-based model was performed to analyze the potential associations between each drug–target pair with these features.

**Figure 4:**
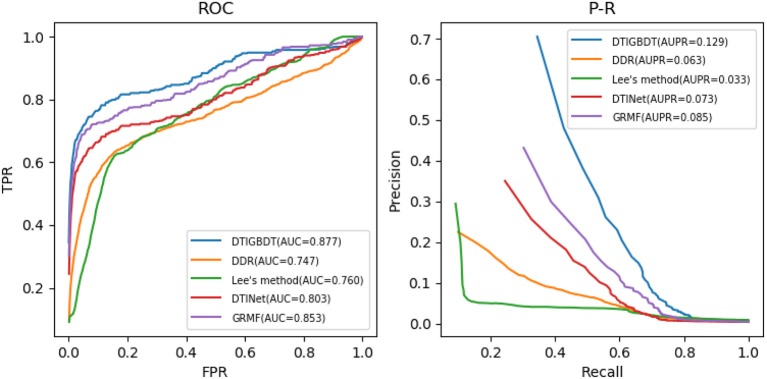
ROC curves and precision–recall curves of DTI prediction by different methods.

Several parameters may influence the performance of DTIGBDT, including the restart probability *a*, the number of neighbors *k*, and the regularization parameter λ. The ranges of *a, k*, and λ are set to {0.2,0.4,0.6,0.8}, {10,20,30,40,50}, and {0.01,0.1,1,10}, respectively. The results of cross validation showed that our method achieves the best performance when *a* = 0.4, *k* = 30, and λ = 0.1. For fair comparison, the parameters of the other methods were also adjusted to obtain their best performance (*n* = 600, *k* = 5 in DDR; *r* = 0.8 in Lee's method; η = 0.5, *d* = 0.1, *t* = 0.1, *l* = 2 in GRMF; and λ = 1, *r* = 0.8 in DTINet). The performance of each method was obtained by using the optimum parameters in each case. The ROC curves and precision–recall curves of all these methods are shown in Figure 4.

DTIGBDT achieves the best performance (AUC = 0.877, AUPR = 0.129), and it achieves 2.3% higher AUC and 4.3% higher AUPR than the second-best method, GRMF. Comparing to DTINet, DTIGBDT achieves 7.3% higher AUC and 5.7% higher AUPR. Both GRMF and DTINet have applied a low-rank model to reduce the dimension of the drug features and target features. However, a great deal of valuable information may be lost in this process. Lee's method does not perform well because it only used the same quantities of negative samples as that of the positive samples to train the *k*-nearest-neighbor model and most of the negative samples were discarded. The AUC and AUPR of DTIGBDT are 11.6% and 9.7% higher than Lee's method, respectively. DDR shows the worst performance because its' prediction model fails to accurately estimate the interaction scores, and the AUC and AUPR of DTIGBDT are 12.9 and 6.6% higher than DDR, respectively. The superior performance of DTIGBDT is mainly due to our model based on GBDT that completely exploits all the negative samples.

We performed a paired *t*-test to evaluate whether DTIGBDT's performance (AUC and AUPR) is significantly better than that of other methods (Ruxton, 2006). The *p*-values are listed in Table 1. These statistical results show that DTIGBDT achieves a significantly better performance than all other methods at the significance level 0.05.

**Table 1:** *P*-values between DTIGBDT and other methods based on AUCs and AUPRs.

	**DDR**	**Lee's method**	**DTINet**	**GRMF**
*P*-values based on AUC	2.3732e-04	5.1773e-08	4.9252e-03	4.3850e-02
*P*-values based on AUPR	7.5153e-14	8.0531e-23	9.8030e-15	6.1235e-09

**Figure 5:**
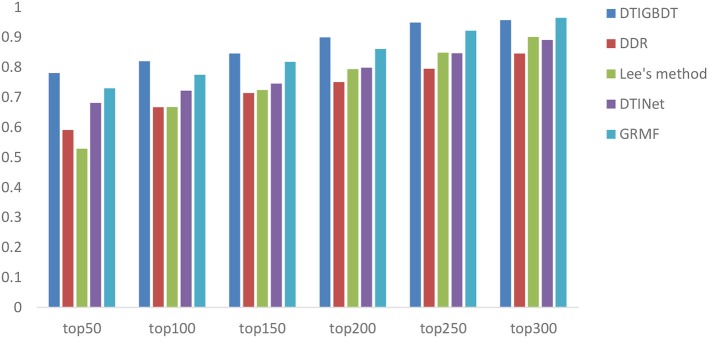
The average recalls across all the tested drugs at different top *k*-values.

A higher recall value for the top *k* reveals that more positive samples are identified successfully. The average recall values of all drugs, for various *k* values, are shown in Figure 5. DTIGBDT outperforms the other methods at each of the *k* cutoffs, and successfully identified 78.1% of the positive samples in the top 50, 82.1% in the top 100, and 90.9% in the top 200. GRMF achieved the second-best performance, for which identified 73.1% in the top 50, 77.5% in the top 100, and 86.1% in the top 200. DTINet identified 68.1% in the top 50, 72.2% in the top 100, and 79.9% in the top 200. Lee's method identifies 52.9% in the top 50, 66.8% in the top 100, and 79.4% in the top 200, which is worse than DTINet but better than DDR. DDR suffers the worst performance, which only identified 59.1% positive samples in the top 50, 71.4% in the top 100, and 75.1% in the top 400.

### Case Studies on Five Drugs

To demonstrate the ability of DTIGBDT to discover potential DTIs, we used it to predict novel drug-related targets. We performed DTIGBDT for all the drugs. All the known DTIs were used to train the model, and the prediction results are listed in Supplementary Table 1. In particular, we executed case studies on five drugs, including *Quetiapine, Clozapine, Olanzapine, Aripiprazole*, and *Ziprasidone*. The top-ranked five candidate targets for each drug were collected and listed in Table 2. To confirm these novel interactions, we consulted several reference databases and the biomedical literature to support them.

**Table 2:** Top-ranked five candidates of five drugs.

**Drug name**	**Rank**	**Target name**	**Evidence**
Quetiapine	1	*GABRA1*	DrugBank, KEGG
	2	*SLC6A4*	literature (Sugawara et al., 2015)
	3	*KCNH2*	literature (Hong et al., 2018)
	4	*PTGS1*	DrugBank
	5	*SCN5A*	literature (Serge and Charles, 2008)
Clozapine	1	*GABRG3*	KEGG, CheMBL
	2	*GABRR2*	DrugBank
	3	*GABRR1*	DrugBank
	4	*GABRG2*	KEGG
	5	*GABRA1*	CheMBL
Olanzapine	1	*GABRG3*	KEGG, UniProt
	2	*GABRB2*	KEGG
	3	*GABRR2*	DrugBank
	4	*GABRA4*	UniProt
	5	*GABRB3*	Literature (Filatova et al., 2017)
Aripiprazole	1	*GABRA1*	KEGG, DrugBank
	2	*GABRA3*	KEGG, CheMBL
	3	*GABRG3*	KEGG
	4	*GABRB3*	KEGG
	5	*GABRD*	KEGG, DrugBank
Ziprasidone	1	*GABRA1*	KEGG, DrugBank
	2	*GABRG1*	KEGG
	3	*GABRD*	KEGG, DrugBank
	4	*GABRR2*	KEGG
	5	*GABRB1*	KEGG, DrugBank

*The novel DTIs are proved by other existing evidence (public databases or literature) and the supporting databases are listed in the evidence*.

DrugBank (Wishart et al., 2017) is a database with annotated cheminformatics resources which combines detailed drug data with target information. As shown in Table 2, 10 of the 25 novel interactions were reported in DrugBank, which confirms the drugs were indeed interacted with the targets. CheMBL (Gaulton et al., 2016) contains the binding and functional information of drug-like bioactive compounds and the information of their binding targets. Three of the 25 interactions were contained in CheMBL, indicating that these drugs can interact with their candidate targets. KEGG (Kanehisa and Goto, 2000) is another useful database dealing with genomes, biological pathways, drugs, and chemical substances. There are 15 interactions that can be found in KEGG, which suggests the expression of the genes can be upregulated or downregulated by the drugs. For example, the drug *Aripiprazole* can act as a potentiator to enhance the expression of the target gene *GABRA1* in combination with another drug *Phenobarbital*.

In addition, a database named UniProt (Consortium, 2014), which collects the protein sequence and function information from research literature, is used to find whether a drug can interact with a specific target; this database includes two interactions. Specifically, the expression of two target genes, *GABRG3* and *GABRA4*, can be reduced by drug *Olanzapine* to inhibit the activity of extracellular ligand-gated ion channels.

Finally, four novel interactions, which are labeled with “literature,” were confirmed by some of the published literature that can be found in PubMed (McEntyre and Lipman, 2001). These drugs were confirmed that they can enhance or inhibit the expressions of their candidate genes. For instance, Sugawara et al. found that drug *Quetiapine* can decrease the DNA methylation level of the promoter region of the gene *SLC6A4* (Sugawara et al., 2015). Case studies suggests that DTIGBDT has powerful ability to discover the potential drug-interacted targets.

## Conclusions

In this paper, we proposed a novel method, DTIGBDT, for predicting the target genes that interact with drugs. We incorporated topological information from the heterogeneous interaction network, and the feature vectors between the drug–target pairs were constructed based on the path categories. A GBDT-based model was constructed for predicting candidate target genes, and it can mitigate the impact of class imbalance by completely exploiting the negative samples. The results of 5-fold cross-validation experiments confirm the superiority of DTIGBDT for DTI prediction. The case studies on five drugs further prove the ability of our model to discover the potential interactions. Therefore, DTIGBDT is a powerful tool which may provide reliable candidate target genes for subsequent identification of actual drug–target interactions with wet-lab experiments. In the future, we will develop our methods on parallel platforms (Zou et al., 2013; Guo et al., 2018) for handling the big data problem.

## Data Availability

All datasets analyzed for this study are included in the manuscript and the Supplementary Files.

## Author Contributions

PX and CS conceived the prediction method. PX, CS, and YY they wrote the paper. CS and TS developed the computer programs. TZ and YD analyzed the results and revised the paper.

### Conflict of Interest Statement

The authors declare that the research was conducted in the absence of any commercial or financial relationships that could be construed as a potential conflict of interest.

## Supplementary Material

Supplementary Table 1 Potential candidate target genes interacted with 549 drugs.
